# Bias-Optimized Hydrogen Sensing in a Mo-Electrode Pd/SnO_2_ Thin-Film Sensor with Integrated Microheater

**DOI:** 10.3390/s26041262

**Published:** 2026-02-14

**Authors:** Dong-Chul Park, Yong-Kweon Kim

**Affiliations:** 1 Department of Electrical and Computer Engineering, Seoul National University, Seoul 08826, Republic of Korea; dcpark@dongyang.ac.kr; 2 Department of Semiconductor and Electronic Engineering, Dongyang Mirae University, Seoul 08221, Republic of Korea

**Keywords:** hydrogen gas sensor, SnO_2_ thin film, Pd catalyst, Mo microheater, Mo electrode, Mo temperature sensor, limit of detection

## Abstract

Hydrogen is a key energy carrier for fuel cell vehicles and hydrogen energy systems. However, its colorless and odorless nature, combined with a wide flammability range, poses significant safety risks in the event of leakage. Accordingly, compact and reliable hydrogen sensors capable of low-ppm detection at moderate operating temperatures are essential for early-stage safety monitoring. In this study, a bias-optimized hydrogen gas sensor based on a Pd-functionalized SnO_2_ thin film with Mo electrodes and an integrated microheater is designed, fabricated, and systematically characterized. The sensor employs a Mo-based vertical microheater and a multilayer thermal insulation stack, enabling thermally efficient and stable operation at 250–280 °C with low power consumption. The electrical and sensing properties of the SnO_2_ layer are optimized by controlling the oxygen partial pressure during reactive sputtering and post-deposition annealing. The Pd catalytic layer promotes hydrogen dissociation and spillover, resulting in pronounced resistance modulation through surface redox reactions and interfacial charge transport effects. By systematically optimizing the sensing bias voltage, a clear trade-off between sensitivity enhancement and electrical noise is identified, which allows stable and repeatable operation in the low-ppm regime. The sensor response follows a power-law dependence on hydrogen concentration, and an automated measurement platform is employed to evaluate repeatability and statistical performance. Based on baseline noise analysis and concentration-dependent resistance variation, a limit of detection of approximately 6.4 ppm is achieved. Furthermore, a concentration-normalized figure of merit that combines response magnitude and concentration dependence is introduced to quantitatively assess low-concentration hydrogen sensing performance. These results demonstrate that the proposed Mo-electrode Pd/SnO_2_ thin-film sensor, enabled by bias-optimized operation and integrated thermal control, provides a robust and scalable platform for safety-critical hydrogen leak detection.

## 1. Introduction

International safety regulations for hydrogen fuel systems, such as UN GTR No. 13 [1] and ISO 23273 [2], require that hydrogen concentrations remain below the lower explosive limit (LEL) of approximately 4 vol % [3]. Although these regulations mandate hydrogen leak detection and mitigation systems, they do not specify explicit sensor detection thresholds. From a sensor performance perspective, standardized evaluation procedures, including SAE J3089 [4], emphasize detection limit, sensitivity, and response time under realistic operating conditions. Moreover, experimental studies have reported that hydrogen concentrations on the order of several tens of ppm can arise from permeation and micro-leakage in parked fuel cell vehicles [5]. These observations indicate that effective hydrogen safety systems require sensors capable of detecting hydrogen at ppm-level concentrations, well below the LEL, to ensure sufficient response time for safety-critical actions [6].

Among various hydrogen sensing technologies, metal-oxide semiconductor (MOS) sensors have been widely investigated due to their high sensitivity, simple device structure, and compatibility with microfabrication processes [7,8,9,10,11,12]. In particular, SnO_2_-based resistive sensors are attractive for compact and integrated sensing platforms. Nevertheless, conventional SnO_2_ sensors typically require elevated operating temperatures to activate surface reactions, resulting in high power consumption and limiting their applicability in portable or energy-constrained systems. In addition, achieving reliable low-concentration detection with minimal baseline drift and good reproducibility remains challenging, especially when device-level integration is pursued.

To enhance the sensing performance of SnO_2_-based hydrogen sensors, catalytic surface modification and microheater integration have been extensively explored. Palladium (Pd) is known to efficiently dissociate hydrogen molecules and promote spillover of active hydrogen species onto the SnO_2_ surface, thereby enhancing surface reactions and resistance modulation [13,14,15,16]. Despite these advantages, previously reported Pd/SnO_2_ sensors often exhibit trade-offs among operating temperature, sensitivity normalization, and practical device integration [17,18,19,20,21,22,23]. In particular, thin-film sensors with integrated microheaters frequently suffer from limited thermal efficiency and reduced concentration-normalized performance compared to bulk or externally heated structures [24].

In this work, a microheater-integrated hydrogen gas sensor based on a sputtered SnO_2_ thin film and a Pd catalytic layer is proposed to address these challenges. The electrical properties of the SnO_2_ sensing layer are systematically tailored by controlling the oxygen partial pressure during sputtering and post-annealing conditions, enabling stable baseline resistance and enhanced low-ppm sensitivity. To achieve thermally efficient and cost-effective operation, a Mo-based microheater combined with a multilayer thermal insulation structure is employed, providing stable operation at moderate temperatures (270–300 °C) with reduced power consumption. Through comprehensive device design, fabrication, and quantitative performance evaluation, this study aims to demonstrate a practical and scalable hydrogen sensing platform suitable for safety-critical hydrogen leak detection applications.

## 2. Device Fabrication

### 2.1. Device Structure

**Figure 1:**
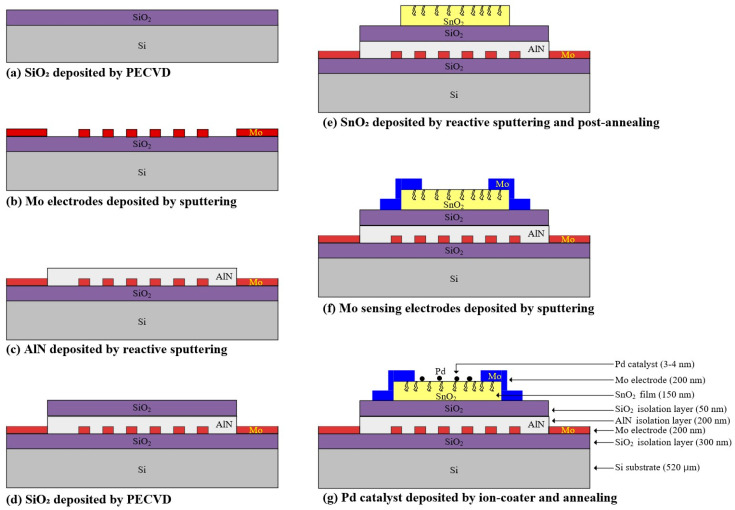
Fabrication process flow and cross-sectional structure of the Pd/SnO_2_ hydrogen sensor with integrated Mo electrodes, microheater, and temperature sensor.

Figure 1 illustrates the fabrication sequence and the vertical structure of the microheater-integrated Pd/SnO_2_ hydrogen sensor. The device is based on a vertically stacked architecture in which a Mo-based microheater and temperature sensor are integrated beneath the SnO_2_ sensing layer. This configuration is designed to achieve efficient localized heating, stable temperature control, and reliable low-ppm hydrogen sensing.

The fabrication process starts with the deposition of a 300 nm SiO_2_ isolation layer on a silicon substrate, as shown in Figure 1a. This layer electrically isolates the device from the substrate and provides a stable base for subsequent high-temperature operation. As shown in Figure 1b, a 200 nm-thick Mo layer is then deposited and patterned to form both the microheater and the temperature-sensing electrodes. Mo is selected due to its high melting point, good thermal stability, and compatibility with repeated thermal cycling at operating temperatures of 270–300 °C. To control heat flow and suppress electrical interference between the heater and the sensing layer, as shown in Figure 1c, a 200 nm AlN layer provides high thermal conductivity while maintaining excellent electrical insulation, allowing heat generated by the microheater to be efficiently delivered to the sensing region. An additional 50 nm SiO_2_ layer is deposited on top of the AlN layer, as shown in Figure 1d. This layer ensures complete electrical isolation between the heater structure and the SnO_2_ sensing layer. The SnO_2_ sensing film with a thickness of 150 nm is then deposited and patterned, as shown in Figure 1e. Post-deposition annealing is performed at 400 °C in a furnace under an atmosphere of 5% O_2_ balanced in N_2_ to improve the crystallinity and stabilize the electrical properties of the sensing layer. After defining the sensing layer, Mo sensing electrodes are deposited and patterned on top of the SnO_2_ film, as shown in Figure 1f. These electrodes form a lateral conduction path through the SnO_2_ layer, enabling resistance-based hydrogen detection. Finally, an ultrathin Pd catalytic layer with a thickness of 3–4 nm is deposited on the SnO_2_ surface and subsequently annealed at 270 °C for 25 min in a nitrogen atmosphere using a furnace, as shown in Figure 1g. The Pd catalyst promotes hydrogen dissociation and spillover onto the SnO_2_ surface, thereby enhancing sensitivity, particularly in the low-ppm concentration range.

**Figure 2:**
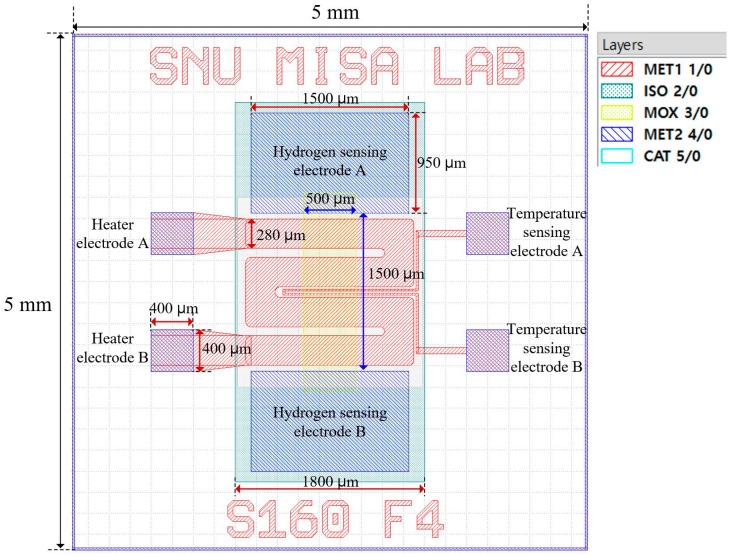
Photomask layout and key geometrical dimensions of the Pd/SnO_2_ hydrogen sensor with integrated Mo electrodes, microheater, and temperature sensor. The texts “SNU MISA LAB” and “S160 F4” are chip identification markings patterned in the MET1 layer and do not contribute to the sensing functionality of the device.

Figure 2 presents the photomask layout and key geometrical parameters of the fabricated sensor. The overall chip size is 5 mm × 5 mm, which is suitable for wafer-level fabrication and subsequent wire bonding and testing.

The active sensing region is located at the center of the device and has dimensions of 1500 μm × 500 μm. This region corresponds to the overlap area between the SnO_2_ sensing layer and the Mo sensing electrodes, where hydrogen adsorption induces resistance modulation. Two Mo-based hydrogen sensing electrodes, labeled electrode A and electrode B, are positioned above and below the sensing region. This configuration establishes a lateral current flow through the SnO_2_ thin film. Beneath the sensing region, a serpentine-shaped Mo microheater is integrated to provide localized Joule heating. The effective heater length is approximately 5800 μm, which is designed to match the sensing length and ensure uniform temperature distribution across the active area. Adjacent to the microheater, Mo-based temperature-sensing electrodes are integrated to enable real-time monitoring of the local temperature. This on-chip temperature feedback allows precise control of the sensor operating temperature and compensates for uncertainties associated with external temperature measurements. The photomask set consists of five functional layers, including the heater and temperature sensor layer, isolation layers, sensing layer, sensing electrode layer, and catalytic layer.

### 2.2. Electro-Thermal Analysis of the Microheater

The thermal behavior of the Mo microheater was evaluated using electro-thermal simulation and simplified analytical estimation as a first-order feasibility assessment for hydrogen sensing operation in the target temperature range.

Electro-thermal simulations were performed using COMSOL Multiphysics (version 5.5) under steady-state conditions based on a simplified heater-on-silicon model. The entire silicon substrate was assumed to be fully exposed to ambient air without any additional heat sink. The silicon substrate had a thickness of 520 μm and an effective heat dissipation area of 25 mm^2^. Heat transfer to the surrounding environment was modeled using a convective heat transfer coefficient of *h* = 40 W·m^−2^·K^−1^, corresponding to weak forced convection under a gas flow of approximately 500 sccm, while the ambient temperature was fixed at 25 °C [24,25].

The electrical resistance of the microheater was set to 200 Ω, which is consistent with the measured sheet resistance of 9.2 Ω/sq for a 200 nm Mo film and the designed heater geometry with a length of 5800 μm and a width of 280 μm. Joule heating power, calculated from the applied voltage and heater resistance, was used as the heat source in both the numerical simulation and the analytical estimation.

**Figure 3:**
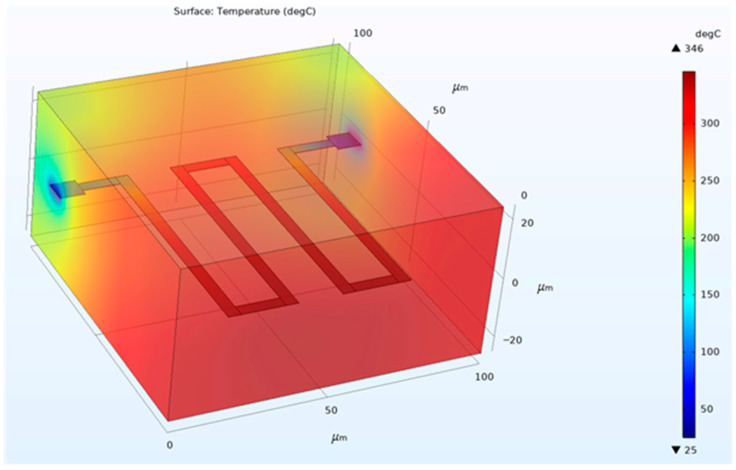
Simulated steady-state temperature distribution of the Mo serpentine microheater.

Figure 3 shows that Joule heating is localized along the serpentine microheater, producing the highest temperature in the central region. Under these conditions, the simulated maximum steady-state temperature reaches approximately 346 °C. This result is consistent with the analytical estimation based on an equivalent thermal resistance model and confirms that the designed microheater can achieve the operating temperature range required for SnO_2_-based hydrogen sensing. Although the detailed multilayer insulation and sensing stack were not explicitly included in the model, the agreement between simulation and analytical estimation provides a reliable first-order validation of the thermal feasibility of the proposed microheater-integrated sensor platform.

### 2.3. XPS Analysis of Post-Annealed SnO_2_ Thin Films

X-ray photoelectron spectroscopy (XPS) analysis of the annealed SnO_2_ thin films was conducted using an AXIS SUPRA (Kratos Analytical Ltd., Manchester, UK) to investigate elemental composition and chemical bonding states.

**Figure 4:**
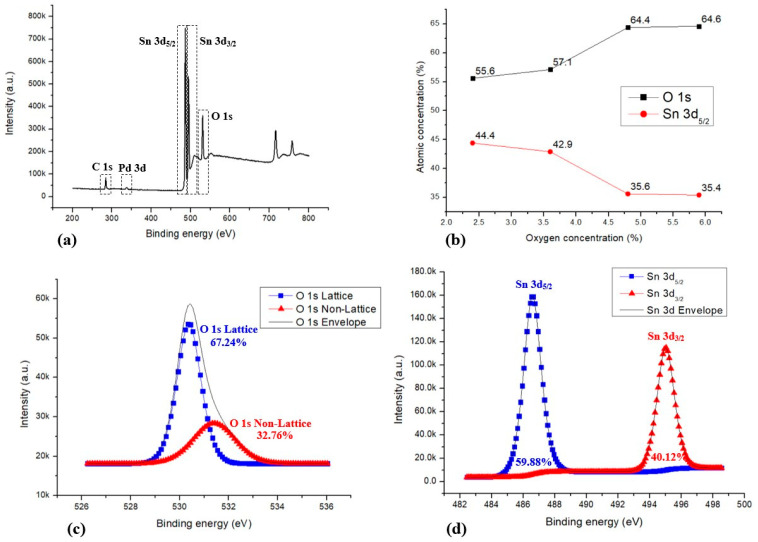
XPS analysis of the SnO_2_ thin films measured after annealing. (**a**) Wide-scan XPS spectrum showing the presence of Sn, O, and Pd. (**b**) Atomic concentration of O 1s and Sn 3d_5/2_ as a function of oxygen concentration relative to Ar during sputtering. (**c**) High-resolution O 1s spectrum after annealing, deconvoluted into lattice oxygen and non-lattice oxygen-related components. (**d**) High-resolution Sn 3d spectrum after annealing, showing the stabilized Sn 3d_5/2_ and Sn 3d_3/2_ components.

Figure 4a shows the wide-scan XPS spectrum, confirming the presence of Sn and O as the dominant elements, together with a weak Pd signal originating from the ultrathin catalytic layer. No significant impurity-related peaks are observed, indicating good chemical purity after annealing.

Figure 4b summarizes the atomic concentrations of O 1s and Sn 3d_5/2_ as a function of the oxygen concentration during the SnO_2_ thin-film deposition process. With increasing O_2_/Ar ratio, the oxygen atomic fraction increases while the tin fraction decreases correspondingly. Above an O_2_/Ar ratio of approximately 5%, the atomic composition gradually converges toward the stoichiometric O:Sn ≈ 2:1 ratio. In addition, the stable Sn 3d_5/2_–Sn 3d_3/2_ doublet indicates that the tin oxidation state is largely stabilized after annealing [26,27,28]. The detailed oxygen bonding states after annealing are shown in Figure 4c. The O 1s spectrum is deconvoluted into lattice oxygen and non-lattice oxygen-related components. The dominant contribution from lattice oxygen indicates an improved oxygen bonding order and surface stoichiometry of the SnO_2_ film after annealing.

Figure 4d shows the corresponding Sn 3d core-level spectrum after annealing. The spectrum consists of the Sn 3d_5/2_ and Sn 3d_3/2_ doublet, and the relative contribution of these components indicates that the tin oxidation state is largely stabilized after annealing. Together, the O 1s and Sn 3d results suggest that post-annealing primarily improves oxygen-related bonding environments rather than inducing a substantial change in the tin oxidation state.

### 2.4. Optimization of SnO_2_ Sputtering and Annealing Conditions

The sputtering and post-annealing conditions of the SnO_2_ sensing layer were optimized by analyzing plasma stability and the resulting electrical properties of the deposited films. Stable plasma operation is essential to ensure reproducible film stoichiometry and uniform electrical characteristics required for reliable hydrogen sensing.

**Figure 5:**
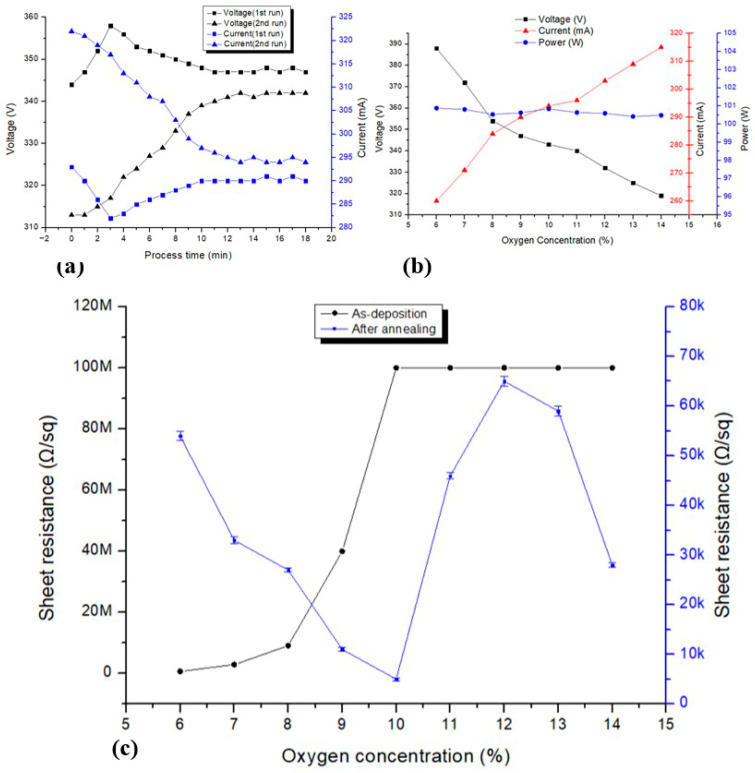
(**a**) Temporal evolution of discharge voltage and current during SnO_2_ sputtering, showing plasma stabilization after an initial transient period. (**b**) Discharge voltage, current, and power as a function of oxygen concentration. (**c**) Sheet resistance of SnO_2_ thin films as a function of oxygen concentration before and after annealing. Error bars indicate the standard deviation (300–1050 Ω) of repeated measurements.

As shown in Figure 5a, the discharge voltage and current exhibit an initial transient behavior at the early stage of sputtering, followed by a stable regime after several minutes. This stabilization reflects the establishment of steady plasma conditions and stabilized target surface states, which are necessary for achieving uniform SnO_2_ thin films [26,27]. Figure 5b shows the dependence of discharge voltage and current on oxygen concentration during SnO_2_ sputtering under constant-power operation, where the oxygen concentration is defined relative to the Ar flow. With increasing O_2_ concentration, the discharge voltage decreases while the current increases, indicating changes in plasma impedance associated with target oxidation. Because the sputtering system operates in a constant-power mode, the discharge power remains nearly constant over the investigated oxygen concentration range.

The electrical properties of the SnO_2_ thin films are summarized in Figure 5c. In the as-deposited state, the sheet resistance increases with oxygen concentration due to the suppression of oxygen-vacancy-related charge carriers. After post-annealing at 400 °C for 1 h in 5% O_2_ balanced in N_2_, the sheet resistance decreases significantly for films deposited at intermediate oxygen concentrations. This behavior is attributed to enhanced carrier mobility associated with partial recovery and redistribution of oxygen vacancies, together with recrystallization at SnO_2_ grain boundaries. In contrast, films deposited under excessively oxygen-rich conditions maintain high resistance even after annealing. These results indicate the existence of an optimal oxygen concentration window that yields stable and suitable electrical properties for hydrogen sensing applications [29].

For oxygen concentrations above 10% in the as-deposited state, the sheet resistance reached the upper measurement limit (≈100 MΩ) of the four-point probe system (CMT-SR2000N, Advanced Instrument Technology (AIT), Suwon, Republic of Korea). Accordingly, an oxygen concentration of 11% was selected as the optimal sputtering condition. Although films with lower resistance exhibit high intrinsic reactivity, their small baseline resistance results in limited dynamic resistance modulation. In contrast, films deposited beyond the resistance inflection point provide a more balanced trade-off between baseline resistance and response amplitude, making them more suitable for dynamic hydrogen sensing.

### 2.5. Morphological and Compositional Characterization

**Figure 6:**
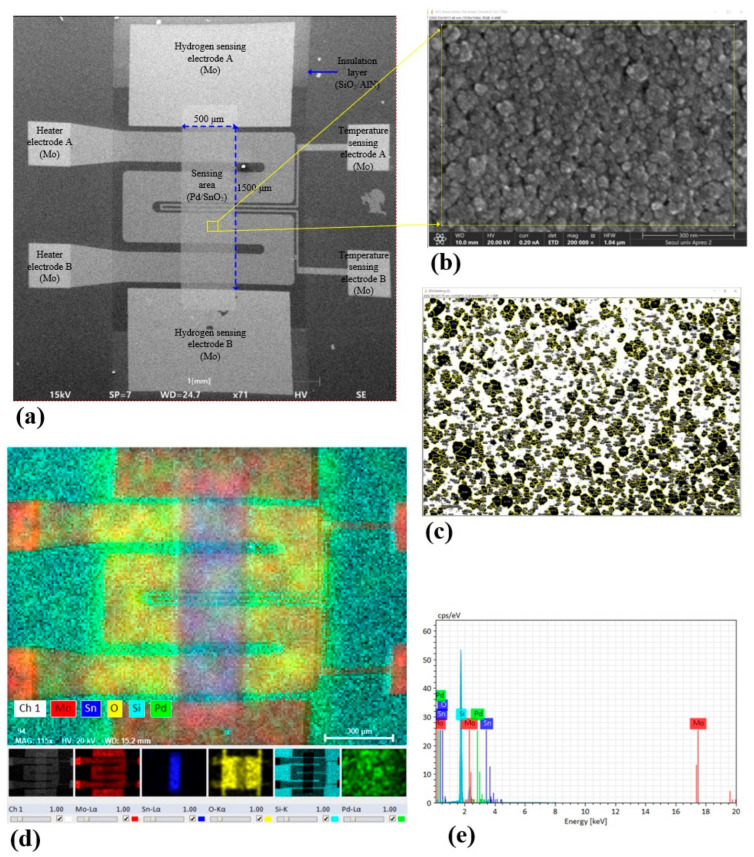
Morphological and compositional characterization of the microheater-integrated Pd/SnO_2_ hydrogen sensor. (**a**) Low-magnification SEM image showing the overall device layout. (**b**) High-magnification SEM image (200,000×) of the SnO_2_ sensing surface after annealing. (**c**) Grain boundary extraction and equivalent circle diameter (ECD) analysis using ImageJ. (**d**) EDS elemental mapping of the sensing region. (**e**) Corresponding EDS spectrum confirming the elemental composition.

Figure 6 presents the morphological and compositional characterization of the microheater-integrated Pd/SnO_2_ hydrogen sensor. As shown in Figure 6a, the low-magnification SEM image confirms that the Mo microheater, hydrogen sensing electrodes, and temperature-sensing electrodes are successfully integrated with well-defined geometry. The Pd/SnO_2_ sensing region is precisely located between the heater lines and electrically isolated by the SiO_2_/AlN insulation layers, ensuring effective thermal coupling and stable device operation.

A high-magnification SEM image of the SnO_2_ sensing surface after annealing is shown in Figure 6b. The surface exhibits a dense fine-grained morphology composed of uniformly distributed nanoscale features. To quantitatively evaluate the grain structure, grain boundary extraction and statistical analysis were performed using the ImageJ software (version 1.53), as shown in Figure 6c. The grain size distribution was analyzed using the equivalent circle diameter (ECD) method, yielding a mean grain size of 20.7 nm with a standard deviation of 13.6 nm. This result indicates a nanoscale but polydisperse microstructure, which is advantageous for gas sensing due to increased surface area and enhanced charge modulation at grain boundaries.

The elemental composition and spatial distribution of the sensing region were further examined by energy-dispersive X-ray spectroscopy (EDS). As shown in Figure 6d, Sn and O signals are consistently detected within the sensing layer, while Pd is localized in the catalytic region and Mo is confined to the electrode areas. The corresponding EDS spectrum in Figure 6e confirms the presence of Sn, O, Pd, Mo, Si, and Al without detectable impurity elements [30,31]. These results verify that the intended material composition and spatial localization are well preserved after fabrication and annealing [28].

### 2.6. Gas Sensing Measurement Setup

**Figure 7:**
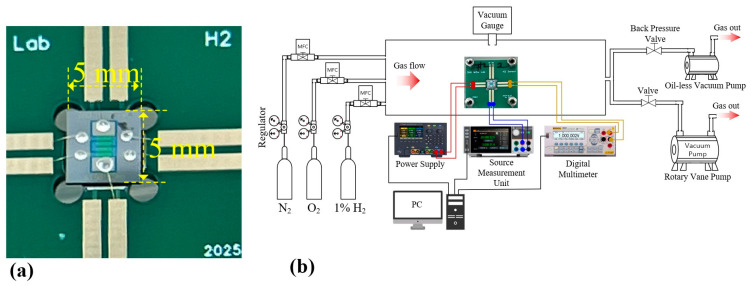
Experimental setup for hydrogen-sensing measurements. (**a**) Photograph of the microheater-integrated hydrogen sensor chip bonded onto the PCB using 353ND epoxy and 25 μm diameter gold wire bonding. (**b**) Schematic diagram of the gas-delivery and electrical measurement system, including mass flow controllers (MFCs), vacuum components, and measurement instruments.

Hydrogen-sensing experiments were conducted using a fully gas-controlled measurement system, as illustrated schematically in Figure 7. A photograph of the actual experimental setup is provided in Figure S1. The gas composition was precisely regulated by three mass flow controllers (MFCs): a 10-sccm MFC for a premixed 1% H_2_/N_2_ source and two 500-sccm MFCs for high-purity N_2_ (5N) and O_2_ (4N). Target hydrogen concentrations were generated by adjusting the flow rate of the 1% H_2_ stream within the range of 0–10 sccm, while compensating the N_2_ and O_2_ flow rates to maintain a constant total flow of 500 sccm.

The sensing chamber with dimensions of 5 cm × 22 cm × 5 cm, corresponding to a volume of approximately 550 cc, was equipped with a Pirani vacuum gauge (CVM211, INFICON InstruTech, LLC, Longmont, CO, USA) and a back-pressure valve to maintain atmospheric pressure during sensing measurements. Using a diaphragm pump in combination with the back-pressure valve, the chamber was operated at a constant pressure of 760 Torr while maintaining a fixed total gas flow rate of 500 sccm. Under this condition, one complete gas exchange occurs approximately every 66 s, which enables rapid stabilization of the gas environment during concentration switching. For initial chamber cleaning and purge procedures, a rotary vane pump was used to evacuate the chamber to a base pressure of approximately 10 mTorr before introducing the measurement gases.

Electrical biasing and resistance measurements were performed using a programmable source measurement unit (NGU401, Rohde & Schwarz GmbH & Co. KG, Munich, Germany), DC power supply (E36312A, Keysight Technologies, Santa Rosa, CA, USA), and a 6½-digit digital multimeter (DM3068, RIGOL Technologies Co., Ltd., Suzhou, China), all interfaced with a PC for automated control and real-time data acquisition. During gas sensing measurements, the SMU was operated in a constant-voltage source mode, applying a fixed bias voltage to the sensing electrodes while measuring the resulting current, from which the sensor resistance was calculated in real time (R = V/I). The digital multimeter was used to continuously monitor the resistance of the on-chip Mo temperature-sensing electrode. Based on the calibrated resistance–temperature relationship, a PC-based negative-feedback control algorithm dynamically adjusted the output voltage of the DC power supply driving the integrated Mo microheater to maintain a constant operating temperature. During sensing measurements, the microheater was typically operated in the temperature range of 270–280 °C, which provided optimal sensitivity and stable baseline characteristics.

The Pd/SnO_2_ sensing layer was biased at a constant voltage using the source measurement unit, and the corresponding resistance change was recorded in real time as the sensing signal. At the target operating temperature, the Mo temperature-sensing electrode exhibited a resistance of approximately 1020 Ω, enabling reliable closed-loop temperature control throughout the sensing experiments.

## 3. Results

### 3.1. Thermal Characterization of the Mo Temperature Sensor and Microheater

**Figure 8:**
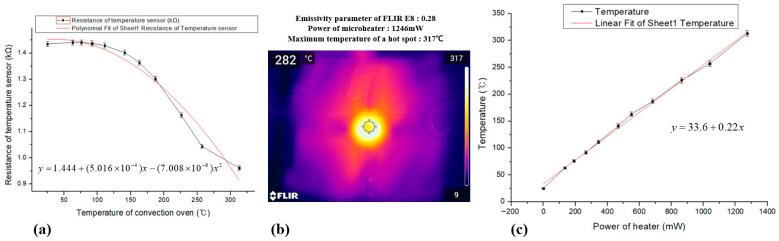
(**a**) Resistance–temperature (R–T) characteristics of the Mo temperature sensor. (**b**) Infrared thermal images of the Mo microheater acquired using a FLIR E8 thermal camera, with emissivity calibrated based on the Mo temperature sensor. (**c**) Steady-state surface temperature of the Mo microheater as a function of Joule heating power, showing a linear temperature–power relationship.

Figure 8 presents the thermal characterization results of the Mo-based temperature sensor and the integrated microheater. To establish an accurate temperature calibration, the resistance of the built-in Mo temperature sensor was first measured in a convection oven. The device temperature was increased stepwise from room temperature to approximately 317 °C, and the corresponding sensor resistance was recorded after thermal stabilization at each step.

As shown in Figure 8a, the Mo temperature sensor exhibits a nonlinear resistance–temperature (R–T) behavior over the measured temperature range. The resistance remains nearly constant or slightly increases at low temperatures, followed by a gradual decrease at higher temperatures, indicating a mixed PTC–NTC characteristic typical of sputtered Mo thin films. The experimental data were accurately fitted using a second-order polynomial function, which was subsequently employed as the calibration curve for real-time temperature estimation during microheater operation [31].

Based on this calibrated temperature sensor, the surface temperature of the Mo microheater was evaluated using infrared thermography, as shown in Figure 8b. Thermal images acquired with a FLIR E8 camera (FLIR Systems, Inc., Wilsonville, OR, USA) under different applied heater powers reveal a localized temperature maximum at the center of the serpentine heater. The emissivity of the heater surface was set to 0.28 using calibration data obtained from the integrated Mo temperature sensor, which allowed a reliable estimation of the absolute surface temperature from infrared thermography. Infrared thermography without calibration using an internal temperature sensor can result in substantial temperature measurement errors. Because the apparent temperature strongly depends on surface emissivity, which is influenced by surface finish and glossiness, large discrepancies can arise in metallic heater structures if emissivity correction is not applied.

Figure 8c summarizes the steady-state Joule heating characteristics of the Mo microheater. At a surface temperature of 280 °C, the power consumption is approximately 1.124 W. This linear relationship confirms the stable and predictable thermal behavior of the microheater, which is essential for reproducible hydrogen-sensing operation under controlled temperature conditions.

### 3.2. Bias Optimization and Dynamic Hydrogen Sensing Behavior

**Figure 9:**
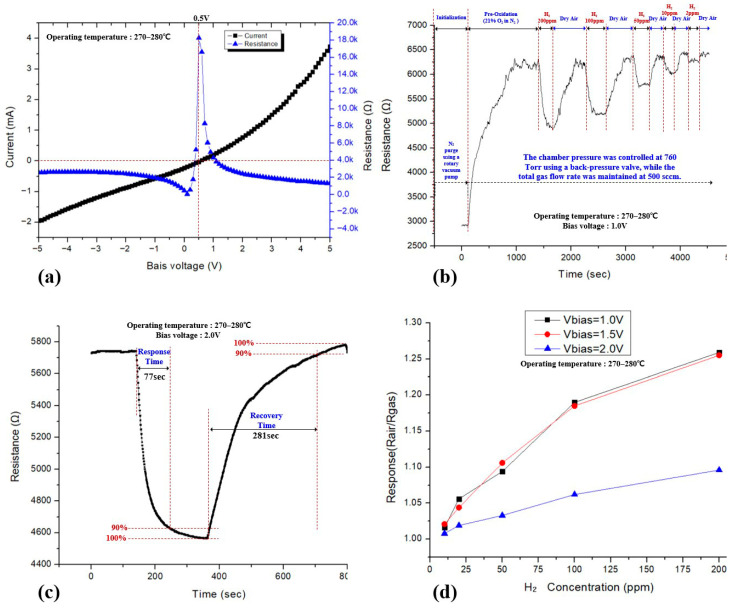
Bias-dependent electrical characteristics and dynamic hydrogen-sensing performance of the Pd/SnO_2_ sensor operated at 270–280 °C. (**a**) Current–voltage (I–V) and resistance–voltage (R–V) characteristics showing a pronounced resistance peak near low bias voltages. (**b**) Dynamic resistance response of the sensor under stepwise hydrogen exposure from 2 to 200 ppm in dry air at a sensing bias of 1.0 V after surface initialization and pre-oxidation. (**c**) Enlarged dynamic response curve used to extract the response time and recovery time, defined as the time required to reach 90% of the total resistance change. (**d**) Sensor response (R_air_/R_gas_) as a function of hydrogen concentration measured at different sensing bias voltages.

Figure 9a shows the bias-dependent electrical characteristics of the Pd/SnO_2_ sensor operated at 270–280 °C. As the applied bias voltage approaches the resistance peak in the R–V characteristic, the sensor resistance increases sharply. In this regime, hydrogen exposure induces strong resistance modulation due to enhanced Schottky barrier modulation at the Pd/SnO_2_ interfaces, resulting in high intrinsic sensitivity. However, operation too close to the resistance peak also leads to increased electrical noise caused by amplified thermal noise, contact resistance fluctuations, and bias-induced instabilities. Therefore, the sensing bias voltage must be carefully optimized to balance sensitivity and signal stability, selecting a bias sufficiently close to the peak to achieve strong resistance modulation while avoiding excessive noise.

Based on this optimization, dynamic hydrogen-sensing measurements were performed at a sensing bias of 1.0 V and an operating temperature of 270–280 °C, as shown in Figure 9b. The sensor exhibits clear and repeatable resistance decreases upon stepwise exposure to hydrogen concentrations ranging from 2 to 200 ppm in dry air, as summarized in Table S1. Each hydrogen pulse is followed by a stable recovery after switching back to dry air, indicating reliable catalytic dissociation of hydrogen on the Pd layer and effective modulation of the SnO_2_ surface depletion layer.

To further improve baseline stability and suppress cycle-to-cycle drift, a surface initialization and pre-oxidation procedure was introduced prior to sensing measurements. After N_2_ purging and pre-oxidation in 21% O_2_ balanced in N_2_, the sensor shows a stabilized baseline resistance and highly reproducible responses during repeated hydrogen exposure cycles. This result indicates that proper surface conditioning mitigates residual surface reduction and incomplete desorption effects accumulated during previous sensing operations.

Figure 9c presents the dynamic response characteristics under the optimized operating conditions. The response time and recovery time are approximately 77 s and 281 s, respectively, defined based on the 90% resistance change criterion. These values inherently include the gas exchange time of approximately 66 s required to completely replace the chamber atmosphere under the present flow conditions. The observed asymmetric response–recovery behavior reflects rapid surface reduction induced by hydrogen adsorption and dissociation, followed by a slower re-oxidation process in dry air, which is characteristic of Pd-decorated metal-oxide gas sensors.

Figure 9d summarizes the dependence of the sensor response (R_air_/R_gas_) on hydrogen concentration for sensing bias voltages of 1.0 V, 1.5 V, and 2.0 V. At the optimized bias of 1.0 V, the response increases monotonically with hydrogen concentration over the range of 2–200 ppm, demonstrating consistent and stable sensitivity in the low-ppm regime. In contrast, higher bias voltages result in reduced response magnitude, confirming that bias optimization is critical for maximizing low-concentration hydrogen-sensing performance. At excessively low bias voltages, the response decreases due to increased noise levels, while an optimal sensing performance is achieved in the bias range of 1.0–1.5 V.

Based on the low-concentration sensing characteristics, the limit of detection (LOD) was estimated to quantitatively assess the minimum detectable hydrogen concentration.

(1) $$LOD=\frac{3\sigma }{S}$$

The baseline noise level was characterized by the standard deviation (*σ* = 24.1 Ω) of the sensor resistance, obtained from repeated measurements of two devices over a two-week period under dry-air conditions at the optimized operating point. The low-concentration sensitivity was extracted as *S* = d(Δ*R*)/d*C* = 11.27 Ω/ppm from a linear fit of the response in the 2–20 ppm regime. Using the conventional criterion, *LOD* = 3*σ*/*S* (Equation (1)), the resulting *LOD* was estimated to be approximately 6.4 ppm.

## 4. Discussion

### 4.1. Thermal Stabilization and Measurement Reliability

The proposed Pd/SnO_2_ thin-film hydrogen sensor operates at elevated temperatures of 270–280 °C, where thermomechanical stress arising from mismatched thermal expansion coefficients among the SnO_2_ sensing layer, insulating layers, and Mo electrodes significantly affects device stability. During initial operation without prior conditioning, transient temperature overshoot and baseline resistance fluctuations were observed, resulting in degraded sensing reproducibility.

To mitigate this effect, a thermal burn-in process of approximately 24 h was applied prior to sensing measurements. After burn-in, both the baseline resistance and temperature response became stable, indicating stress relaxation and structural stabilization of the thin-film stack. This result highlights the importance of thermal conditioning for reliable long-term operation of thin-film hydrogen sensors under prolonged high-temperature conditions.

### 4.2. Pd/SnO_2_ Interfacial Barrier Modulation with Preadsorbed Oxygen Ions and Physisorption Contribution

**Figure 10:**
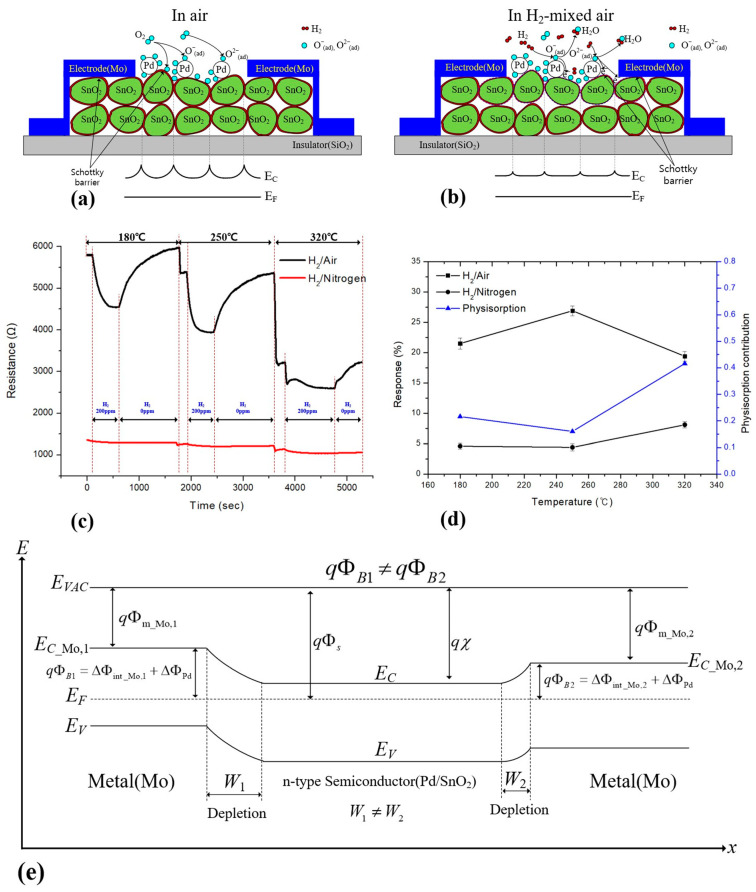
(**a**) Dynamic resistance responses of the Pd/SnO_2_ sensor to 200 ppm H_2_ under air and nitrogen backgrounds at different operating temperatures. (**b**) Temperature-dependent sensing responses in H_2_/air and H_2_/N_2_, together with the estimated physisorption contribution. (**c**) Schematic illustration of oxygen-ion-mediated barrier formation in air, showing enlarged depletion regions and Schottky barriers. (**d**) Schematic of barrier modulation under H_2_-mixed air, where hydrogen-induced reduction and spillover lower the interfacial and grain-boundary barriers. (**e**) Equivalent electrical model and energy band diagram of the Mo-Pd/SnO_2_-Mo structure.

As schematically illustrated in Figure 10a,b, the hydrogen-sensing behavior of the proposed Pd/SnO_2_ device is governed by dynamic modulation of the interfacial Schottky barrier in conjunction with grain-boundary potential barriers, which are strongly influenced by pre-adsorbed oxygen ions and the redox state of the Pd catalyst.

Under air ambient at elevated temperatures (180–320 °C), oxygen molecules are adsorbed and ionized on the Pd surface, forming pre-adsorbed oxygen species (O^−^, O^2−^). These oxygen ions partially oxidize the Pd surface, increasing its effective work function and consequently enhancing the Pd/SnO_2_ Schottky barrier height (ΔΦ_Pd_). Simultaneously, electron depletion at the SnO_2_ grain surfaces and grain boundaries is intensified, leading to a widening of the depletion layers and an increase in the overall sensing resistance, as depicted in Figure 10a [17,18,19].

Upon exposure to hydrogen in air (H_2_-mixed air), Pd acts as an efficient catalyst for hydrogen dissociation. The dissociated hydrogen atoms spill over onto the SnO_2_ surface and react with the pre-adsorbed oxygen ions, producing H_2_O and releasing trapped electrons back into the SnO_2_ conduction band. This reduction process decreases the effective work function of Pd, resulting in a lowered interfacial barrier height (ΔΦ′_Pd_), accompanied by a narrowing of depletion regions at both the Pd/SnO_2_ interface and SnO_2_ grain boundaries (Figure 10b). As a result, electron transport across adjacent grains is significantly enhanced, producing a pronounced decrease in sensing resistance.

To decouple the contribution of chemisorption mediated by oxygen ions from physical adsorption effects, comparative sensing measurements were performed in oxygen-containing air and oxygen-free nitrogen environments, as shown in Figure 10c. In ambient nitrogen, where the formation of pre-adsorbed oxygen ions is suppressed, the sensor still exhibits a measurable but substantially reduced response to hydrogen. This residual response is attributed primarily to physisorption-driven mechanisms, including weak hydrogen adsorption and Pd–H interactions that modulate the local carrier density without involving oxygen-mediated redox reactions.

Figure 10d quantitatively summarizes the temperature-dependent sensing response in air and nitrogen, along with the extracted physisorption contribution. The physisorption contribution remains relatively small compared to the total response in air, particularly at intermediate temperatures (250 °C), where oxygen-ion-mediated barrier modulation dominates. However, at higher temperatures (320 °C), the relative contribution of physisorption increases, indicating that oxygen desorption and reduced surface coverage weaken chemisorption pathways, allowing physisorption-related processes to play a more noticeable role [32].

These results demonstrate that the hydrogen-sensing response of the Pd/SnO_2_ device arises from a synergistic combination of oxygen-ion-mediated chemisorption and physisorption-driven effects. Importantly, the dominant sensing mechanism under practical operating conditions is barrier-controlled conduction, originating from coupled modulation of the Pd/SnO_2_ interfacial Schottky barrier and SnO_2_ grain-boundary potential barriers, while physisorption provides a secondary but non-negligible contribution, particularly at elevated temperatures.

### 4.3. Asymmetric Mo/SnO_2_ Contacts and Bias-Dependent Behavior

The asymmetric, bias-dependent electrical behavior of the sensing resistance arises from unequal Schottky barriers formed at the two Mo/SnO_2_ contacts. Although the sensing electrodes were designed to be geometrically symmetric, minor lithographic misalignment during fabrication resulted in different effective contact areas between the SnO_2_ sensing layer and the opposing Mo electrodes. This leads to non-identical interfacial barrier heights and depletion widths (ΔΦ_int_Mo,1_ ≠ ΔΦ_int_Mo,2_, *W*_1_ ≠ *W*_2_), producing an apparent built-in potential across the device, as illustrated in Figure 10e. Consequently, the net current polarity does not reverse at zero bias.

With increasing applied bias, the relative influence of the two asymmetric Mo/SnO_2_ junctions on the overall resistance changes. At an applied voltage of approximately 0.4–0.5 V, the effective barriers at the two contacts become comparable, resulting in a switching behavior in the current–voltage characteristics. This observation confirms that the sensing resistance is governed not only by Pd/SnO_2_ interfacial reactions related to hydrogen adsorption but also by contact asymmetry at the Mo/SnO_2_ junctions, highlighting sensing-bias optimization as a critical parameter for stable and reproducible hydrogen sensing.

### 4.4. Concentration Exponent and Performance Comparison

The sensing response of metal-oxide gas sensors generally follows a power-law dependence on gas concentration, which can be expressed as shown in Equation (2).

(2) $$kC^{n}=\frac{R_{air}}{R_{gas}}-1$$

where *R_air_* and *R_gas_* denote the sensor resistance in air and hydrogen, respectively; *C* is the hydrogen concentration; and *n* is the concentration exponent extracted from log–log fitting of the measured response.

Based on the power-law relationship described in Equation (2), the concentration-normalized figure of merit was defined as follows:

(3) $$k=\frac{\left(R_{air\;}/\;R_{gas}\;-1\right)\;}{C^{n}}$$

As defined in Equation (3), the *k* normalizes the sensor sensitivity with respect to gas concentration, allowing the intrinsic sensing performance to be evaluated independently of the specific concentration range.

The extracted concentration exponent of the proposed sensor (*n* ≈ 0.78 at 280 °C) lies between diffusion-limited systems (*n* = 0.5–0.7) and strongly reaction-dominated or catalytically amplified systems (*n* = 0.9–1.3). This intermediate *n* value indicates that the sensing behavior is governed by a mixed mechanism, in which Schottky-barrier modulation at the Pd/SnO_2_ interface plays a dominant role rather than bulk diffusion or excessive catalytic amplification. The thin-film geometry and moderate Pd loading effectively enhance interfacial sensitivity while suppressing transport-limited effects.

As summarized in Table 1, previously reported SnO_2_-based hydrogen sensors employing nanofibers, nanoparticles, or nanosheet structures often exhibit relatively large response values, mainly due to porous morphologies, high noble-metal loading, or thick sensing layers. However, these approaches typically require complex fabrication processes or external heaters or lack wafer-level compatibility.

Notably, the integration of a vertical microheater and an on-chip Mo temperature sensor enables precise closed-loop temperature control, significantly improving signal stability and allowing the reliable extraction of low-ppm performance metrics. In contrast to previously reported thin-film sensors that rely on external heating stages or ambient temperature-controlled setups, the proposed device is designed as a fully standalone sensing element, in which the sensing temperature is internally defined and actively regulated. As a result, the sensor performance is effectively decoupled from external thermal disturbances. The proposed device exhibits a comparable or lower response, but with markedly improved reproducibility, simpler fabrication, and enhanced suitability for practical and integrated applications.

This study focuses on evaluating the feasibility of Mo electrodes and their compatibility with Pd/SnO_2_-based hydrogen sensing, rather than providing an exhaustive assessment of gas selectivity. The results demonstrate that Mo electrodes reliably support stable microheater operation, accurate on-chip temperature sensing, and effective hydrogen response when integrated with a Pd-catalyzed SnO_2_ thin film.

While selectivity toward hydrogen over other reducing or interfering gases was beyond the scope of this work, the proposed sensor architecture provides a robust and well-controlled platform for such investigations. Future studies will therefore include systematic selectivity and cross-sensitivity measurements under precisely controlled gas mixtures to further evaluate the practical applicability of the device.

Overall, this discussion clarifies how thermal stabilization, interfacial barrier modulation, and electrode feasibility collectively govern the sensing performance of the proposed Pd/SnO_2_ hydrogen sensor. These insights establish a solid foundation for further optimization of thin-film sensor designs and for extended studies on selectivity and long-term reliability.

## 5. Conclusions

In this work, a Pd/SnO_2_ thin-film hydrogen sensor with an integrated microheater was successfully designed and fabricated to achieve reliable 6.41 low-ppm hydrogen detection under well-controlled thermal conditions. Stable baseline behavior was ensured through thermal conditioning and surface initialization, enabling repeatable resistance modulation upon hydrogen exposure.

By optimizing the sensing bias and operating temperature, the device achieved improved signal-to-noise characteristics at relatively low operating temperatures. The sensing mechanism was clarified to be dominated by Pd/SnO_2_ and Mo/SnO_2_ barrier modulation rather than bulk resistance changes.

Furthermore, a conservative limit of detection defined based on long-term baseline dispersion highlights the intrinsic detectability of the sensing element itself. These results demonstrate that bias optimization and closed-loop temperature regulation are key design parameters for practical, microheater-integrated metal-oxide hydrogen sensors and provide a solid foundation for future studies on selectivity and long-term reliability.

## Figures and Tables

**Table 1:** Comparison with previously reported SnO_2_-based H_2_ sensors.

Material	Morphology	Temp. (°C)	Conc. (ppm)	Response (*s *=* R_air_*/*R_gas_*)	Response/ Recovery Time (s)	Integrated Heater	Ref.
Pd/SnO_2_	Nanowire	300	15,000	~9.5	213/33	None	[20]
Au/SnO_2_	Nanoparticle	400	5000	~48	~40/~170	None	[24]
Pd-SnO_2_	Nanofiber	280	100	8.2	9/140	None	[33]
Pd_1_Ag_0.50_@SnO/SnO_2_	Nanosheets	225	100	81.34	45/682	None	[34]
Pd/SnO_2_	Thin-film	280	200	1.26	77/281	Vertical microheater with temperature sensor	This work

## Data Availability

The data supporting the findings of this study are included within the article. Further inquiries can be directed to the corresponding author.

## Supplementary Materials

The following supporting information can be downloaded at: https://www.mdpi.com/article/10.3390/s26041262/s1, Figure S1: A photograph of the actual experimental setup; Table S1: Gas flow conditions used to generate hydrogen concentrations from 2 to 200 ppm.
