# Supplementary material for: Bias-Optimized Hydrogen Sensing in a Mo-Electrode Pd/SnO2 Thin-Film Sensor with Integrated Microheater
Source: Sensors (Basel). 2026 Feb 14;26(4):1262. doi: 10.3390/s26041262 (PMC12944079; doi:10.3390/s26041262)
Supplement: Supplementary file 1 [file sensors-26-01262-s001.zip › sensors-4122199-supplementary.pdf]

sensors

MDPI

Supplementary Materials

Bias-Optimized Hydrogen Sensing in a Mo-Electrode Pd/SnO_2 Thin-Film Sensor with Integrated Microheater

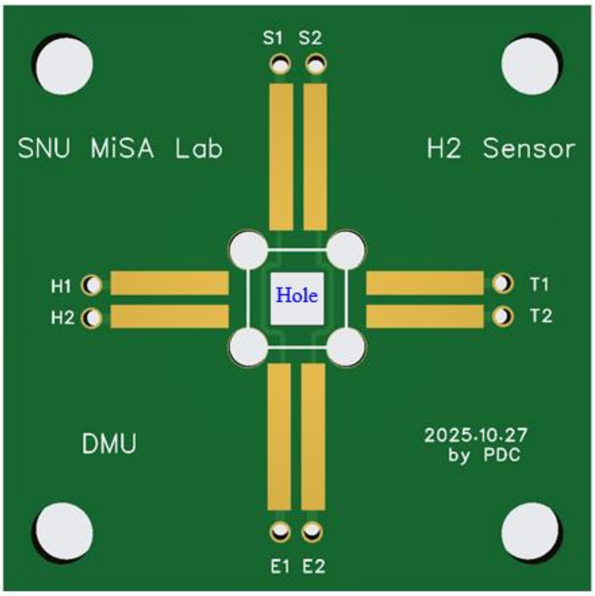

(a) PCB layout for sensor packaging.

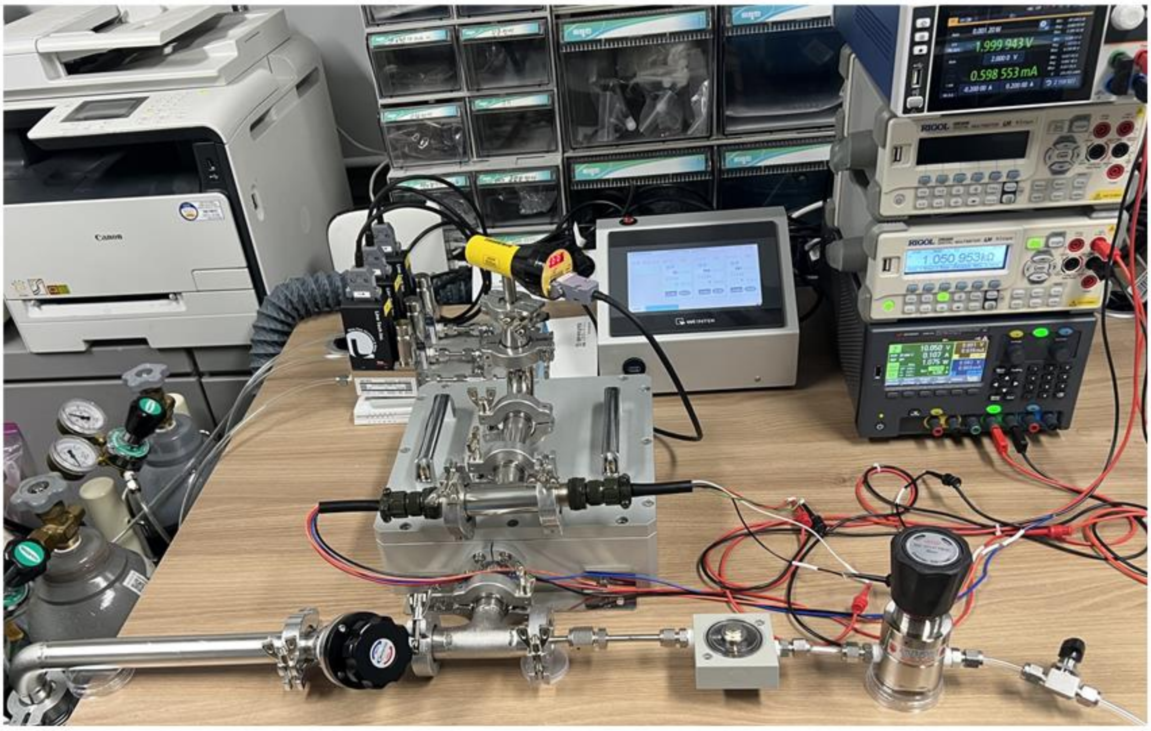

(b) Photograph of assembled hydrogen-sensing setup.

Figure S1. A photograph of the actual experimental setup. (a) Layout of the cus-tom-designed PCB with ENIG surface finish used for sensor mounting and electrical interfacing. (b) Photograph of the assembled experimental setup used for hydro-gen-sensing characterization.

 https://doi.org/10.3390/10.3390/s26041262

Table S1. Gas flow conditions used to generate hydrogen concentrations from 2 to 200 ppm.

H_2 Concentration (ppm)	1% H_2 Flow (sccm)	O_2 Flow (sccm)	N_2 Flow (sccm)	Total Flow (sccm)
**200**	10	99	391	500
**100**	5	102	393	500
**50**	2.5	104	394	500
**20**	1	104	395	500
**10**	0.5	105	395	500
**2**	0.2	105	395	500
**0(Dry Air)**	0	105	395	500

Note: Minor deviations (<0.5 sccm) in the total flow may arise from the resolution of the mass flow controllers (MFCs); however, the total flow was nominally maintained at 500 sccm for all measurements.
